# Influence of contact with schizophrenia on implicit attitudes towards schizophrenia patients held by clinical residents

**DOI:** 10.1186/1471-244X-12-205

**Published:** 2012-11-22

**Authors:** Ataru Omori, Amane Tateno, Takashi Ideno, Hidehiko Takahashi, Yoshitaka Kawashima, Kazuhisa Takemura, Yoshiro Okubo

**Affiliations:** 1 Department of Neuropsychiatry, Nippon Medical School, 1-1-5 Sendagi, Bunkyo-ku, Tokyo, 113-8602, Japan; 2 Department of Psychology, Waseda University, 1-24-1 Toyama, Shinjyuku-ku, Tokyo, 162-8644, Japan; 3 Department of Psychiatry, Kyoto University Graduate School of Medicine, 54 Shogoin-Kawara-cho, Sakyo-ku, Kyoto, 606-8507, Japan; 4 Precursory Research for Embryonic Science and Technology (PRESTO), Japan Science and Technology Agency, 4-1-8 Honcho, Kawaguchi, Saitama, 332-0012, Japan

**Keywords:** Prejudice, Stigma, Implicit association test, Education, Schizophrenia

## Abstract

**Background:**

Patients with schizophrenia and their families have suffered greatly from stigmatizing effects. Although many efforts have been made to eradicate both prejudice and stigma, they still prevail even among medical professionals, and little is known about how contact with schizophrenia patients affects their attitudes towards schizophrenia.

**Methods:**

We assessed the impact of the renaming of the Japanese term for schizophrenia on clinical residents and also evaluated the influence of contact with schizophrenia patients on attitudes toward schizophrenia by comparing the attitudes toward schizophrenia before and after a one-month clinical training period in psychiatry. Fifty-one clinical residents participated. Their attitudes toward schizophrenia were assessed twice, before and one month after clinical training in psychiatry using the Implicit Association Test (IAT) as well as Link’s devaluation-discrimination scale.

**Results:**

The old term for schizophrenia, “Seishin-Bunretsu-Byo”, was more congruent with criminal than the new term for schizophrenia, “Togo-Shitcho-Sho”, before clinical training. However, quite opposite to our expectation, after clinical training the new term had become even more congruent with criminal than the old term. There was no significant correlation between Link's scale and IAT effect.

**Conclusions:**

Renaming the Japanese term for schizophrenia still reduced the negative images of schizophrenia among clinical residents. However, contact with schizophrenia patients unexpectedly changed clinical residents’ attitudes towards schizophrenia negatively. Our results might contribute to an understanding of the formation of negative attitudes about schizophrenia and assist in developing appropriate clinical training in psychiatry that could reduce prejudice and stigma concerning schizophrenia.

## Background

Patients with schizophrenia and their families have suffered greatly from the stigmatizing effects and the educational, vocational, and interpersonal barriers resulting from negative social attitudes toward their conditions. Patients with schizophrenia have tremendous difficulties finding employment and acquiring living quarters, and they suffer from falsely pressed charges for violent crimes by these kinds of stigmatizing effects [1-7]. From this situation has arisen increasing interest in how these negative attitudes towards schizophrenia may interfere with their various abilities and also with their efforts to obtain effective treatment [8,9]. To reduce mental illness-related stigma (particularly regarding schizophrenia), various programs are underway internationally [1,10,11]. Although the view of schizophrenia has been changing according to advances in neurobiological understanding of the disorder, pharmacology and psychosocial treatments [12], prejudice and stigma are still prevalent even among medical professionals, and providing explanations of the biomolecular profile of mental illnesses is not sufficient to solve these problems [13,14]. As part of the attempt to improve this situation, the Japanese Society of Psychiatry and Neurology replaced the old Japanese term for schizophrenia, “Seishin-Bunretsu-Byo” (Mind-Split Disease), with a new term, “Togo-Shitcho-Sho” (Integration Disorder), officially announced at the 12th World Congress of Psychiatry, Yokohama, Japan, 2002. The former term has been said to lead the public to misunderstand and stigmatize individuals with schizophrenia. In western society, the term also implies “split” and is frequently misunderstood as “split personality” [15] or is inappropriately metaphorized [16]. The change of the term in Japan has been disseminated throughout Japanese society and has also attracted worldwide interest [12,17,18]. As mentioned above, the most prevalently held stereotype of people with mental illness is that they are unpredictable and dangerous [6,19]. However, previous studies have revealed that severe mental illness per se does not predict an increased risk of violent behavior and that people with mental illness are far more likely to be victims of crime than perpetrators [20,21]. We assessed the impact of this renaming on implicit stigma associated with schizophrenia using the Implicit Association Test (IAT), reporting that the renaming led to reduced stigma, in that fewer people, at least among non-medical undergraduate students [22], tended to associate the new term with criminality. Information concerning prejudice and stigma, usually gathered using questionnaires, is subject to response bias due to social desirability [2,23-25]. IAT is a method developed in the field of social psychology to assess implicit (unaware, hidden) attitudes by measuring the strength of the associations between mental representations from different categories of objects in memory [26]. The logic of IAT is that the sorting task should be easier, and thus faster, when the two concepts that share a response are implicitly associated. IAT has been successfully employed to assess attitudes associated with homosexuality [27,28], smoking [29], and consumer products [30].Most of the research concerning prejudice and stigma related to schizophrenia is intended to benefit the public, patients and their families. The attitudes of mental health professionals toward schizophrenia are also very important, as they can directly influence treatment outcomes and the quality of life experienced by patients with schizophrenia, as well as have an effect on the general population. Some studies indicated that persons with seemingly more knowledge about mental illness were less likely to endorse stigma and discrimination [22] and that education and exposure lead to a decline in stigmatized attitudes [31,32], whereas other studies indicated that medical professionals’ attitudes were similar to those found among the general public [13,33] or that there was no consensus [34,35]. Exposure to mental illness had a significant influence on the attitudes towards mental illness [36,37]. However, how contact with schizophrenia patients affects the formation of negative attitudes towards schizophrenia at implicit and explicit levels has been poorly understood. Thus, how patients with schizophrenia are viewed by clinical residents after the experience of conducting therapy for schizophrenia should be of considerable interest to medical professionals.Based on these findings, we assessed the impact of the renaming of schizophrenia on the attitudes held by clinical residents, which had already been proven for non-medical undergraduate students as subjects by Takahashi et al. (2009) [22]. Also, as the most interesting theme in this study, we assessed the impact of the contact with schizophrenia patients on these clinical residents to shed some light on the complicated issue of prejudice and stigma, since we considered that prejudice and stigma toward schizophrenia could be based on knowledge and experience of schizophrenia at both implicit and explicit levels, and especially by direct contact with such patients. As subjects we selected clinical residents, for they had just graduated from medical school and so far had presumably had little contact with schizophrenia patients. Then we assessed the change of attitudes toward schizophrenia between before and after clinical training in psychiatry. In the assessment we used IAT according to the previous study [22], as it was designed for both the old diagnostic Japanese term for schizophrenia, “Seishin-Bunretsu-Byo”, and the new diagnostic term “Togo-Shitcho-Sho”. Before beginning their training in psychiatry, clinical residents have of course already completed their classroom course work in schizophrenia, but they have had little experience in terms of direct contact with schizophrenia patients. Then, in their clinical psychiatry training, they experience the application of therapy, and have direct contact with these patients for the first time. Then, by comparing their attitudes toward schizophrenia between before and after their clinical psychiatry training, the significance of having contact with schizophrenia patients became clear, shedding some light on how contact with schizophrenia patients affects the formation of negative attitudes towards schizophrenia at implicit and explicit levels.

## Methods

### Participants

Fifty-one clinical residents in postgraduate medical education (30 males and 21 females, mean age 28.0 (S.D = 4.1) years) participated in this study. In Japan, a 2-year postgraduate training period has been mandatory as a planned introductory internship since 2004 for the improvement of basic medical and practical skills regardless of the future field of specialization. During this period, clinical residents are required to treat a variety of common diseases, and this also includes schizophrenia, mood disorders, and dementia in psychiatry, as part of the care team. Specific psychiatric care teams include physicians having knowledge and experience concerning mental health. This is the first opportunity for clinical residents to treat schizophrenia patients. They meet with schizophrenia patients 5 days a week, with the total time spent with patients being about 160 hours. They assess their symptoms, formulate medication plans, and treat them under the supervision of psychiatrists. They are required to experience three diseases in the psychiatric ward, and the treatment of 1–2 patients with schizophrenia is required during their 1-month clinical training period in psychiatry. This planned introductory internship is mainly based on a biogenetic model and pharmacotherapy. Thus, through this clinical training in psychiatry, they experience the application of therapy for schizophrenia patients for the first time, and at that time they experience direct contact with these patients. The participants were assessed for their attitude toward schizophrenia twice, before and after clinical training of psychiatry, using IAT and questionnaires. The study protocol conformed to the provisions of the Declaration of Helsinki of 1995. The study was approved by the Ethics Committee of Nippon Medical School Hospital, Tokyo, Japan, and after complete explanation of the study, written informed consent was obtained from all participants.

### Measures and procedures

To assess the implicit attitudes towards schizophrenia, the participants first underwent IAT, and then they filled out the questionnaires.

### IAT

**Figure 1:**
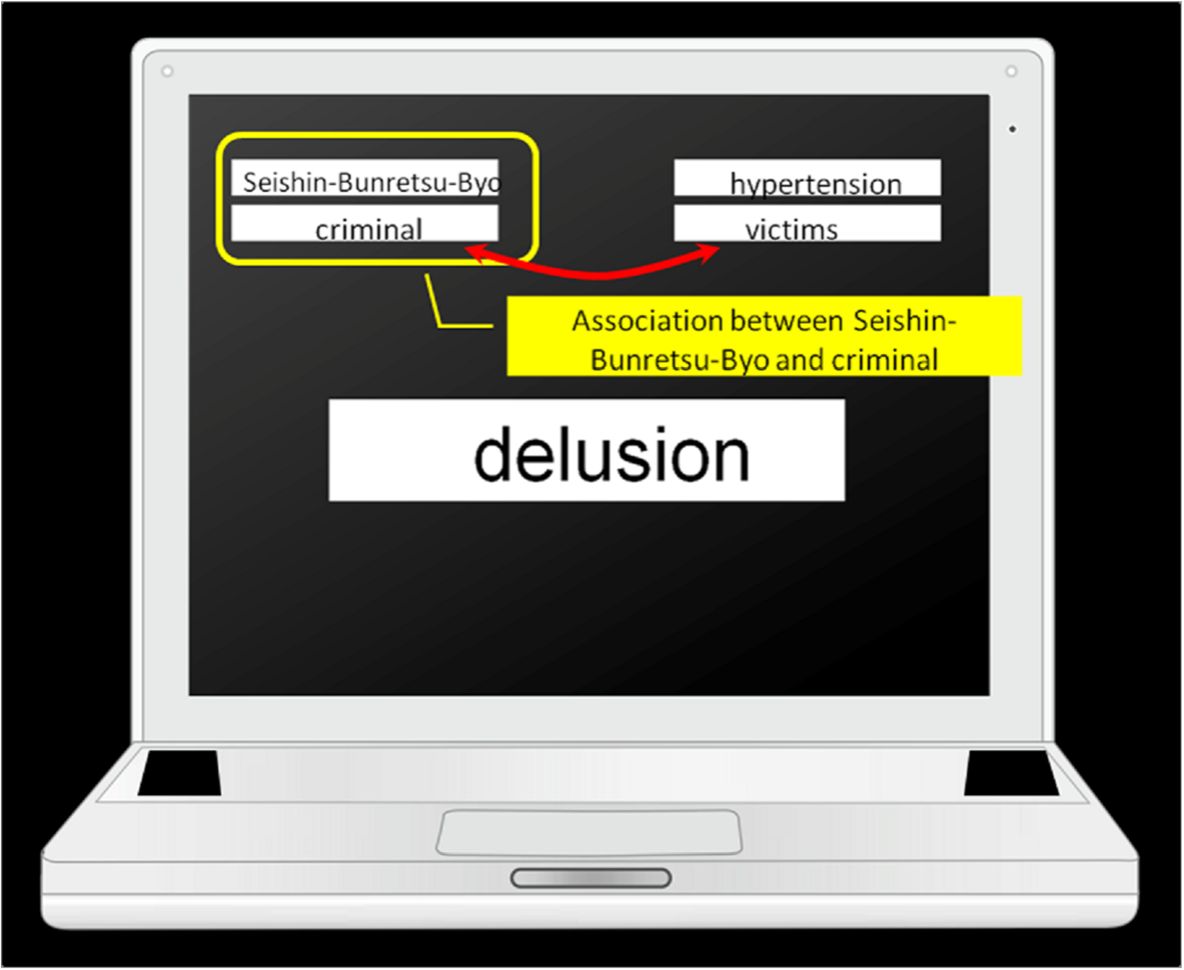
**Ex.) Congruent condition.** Strong implicit associations should lead to fast congruent (CC) and slow incongruent (IC) categorizations. As a result, the IAT effect (reaction time for IC minus CC) provides a measure of the strength of implicit associations.

IAT was performed according to standard procedures to assess the strength of the automatic association between schizophrenia and criminal [26]. IAT tasks corresponded to those of the previous study [22]. While diabetes mellitus was used as a physical chronic illness for comparison in that study, we chose hypertension as a physical illness to contrast schizophrenia, as it is a more common medical condition in Japan. The associations of schizophrenia (both “Seishin-Bunretsu-Byo” version and “Togo-Shitcho-Sho” version) and hypertension with two attributes (criminal and victim) were then assessed. Target words were the same as in the study of Takahashi et al. (2009) [22], and for hypertension the target words were surveyed in the same way. Five words related to hypertension (vessel, sphygmomanometer, antihypertensive, salt, palpitation) were consensually finally selected. Schizophrenia (hallucination, delusion, psychiatry, bizarre, seclusion), criminal (violence, jail, murder, theft, robbery) and victim (disaster, family, accident, casualty, the bereaved) stimuli appeared in the center of the computer screen (see Figure 1). Subjects were asked to respond to a series of items belonging to either the schizophrenia or criminal categories on the left, and those belonging to either the hypertension or victim categories on the right, as rapidly as possible with left- or right-hand key press. In the congruent condition (CC), the concept “schizophrenia” and the attribute “criminal” were paired in the top left corner, while “hypertension” and “victim” were coincidentally paired in the opposite corner. In the incongruent condition (IC), the key assignments for one of the pairs were switched and the same sorting task was completed while pairing “schizophrenia” with “victim” and “hypertension” with “criminal”. Sorting should be easier and faster when the two concepts sharing a response are implicitly associated. Consequently, the IAT effect (reaction time for IC minus CC) showed a measure of the strength of implicit associations. Since negative attitudes toward mental illness are observed in many cultures [38], CC categorizations can be expected to be easier and faster than IC ones. The order of the two versions of IAT was counterbalanced across the subjects.According to Greenwald et al. (1998) [26], response latencies below 300 ms were converted to 300 ms and those above 3,000 ms to 3,000 ms. Latencies were then log-transformed. We analyzed the effect and interaction of term (old term vs. new term), condition (CC vs. IC), and period (before vs. after) using 3-way analysis of variance (ANOVA).

### Questionnaires

To assess the explicit attitudes, participants reported their attitude about mental illness using the Japanese version of the 4-point Link’s devaluation-discrimination scale [39,40]. This consists of twelve 4-point scales ranging from ‘strongly agree = 1' to ‘strongly disagree = 4', yielding a total score from 12 to 48, which included statements such as: ‘Most people would willingly accept a former mental patient as a close friend.’ Link's scale is intended for mental illness in general, not only for schizophrenia, and it assesses the extent to which a person believes that other people will devalue or discriminate against someone with a mental illness. Link’s scale was developed essentially in order to assess respondents' beliefs about mental illnesses, but it is in the form of “Most people think…” rather than “I think …” in order to minimize social desirability bias.

## Results

**Figure 2:**
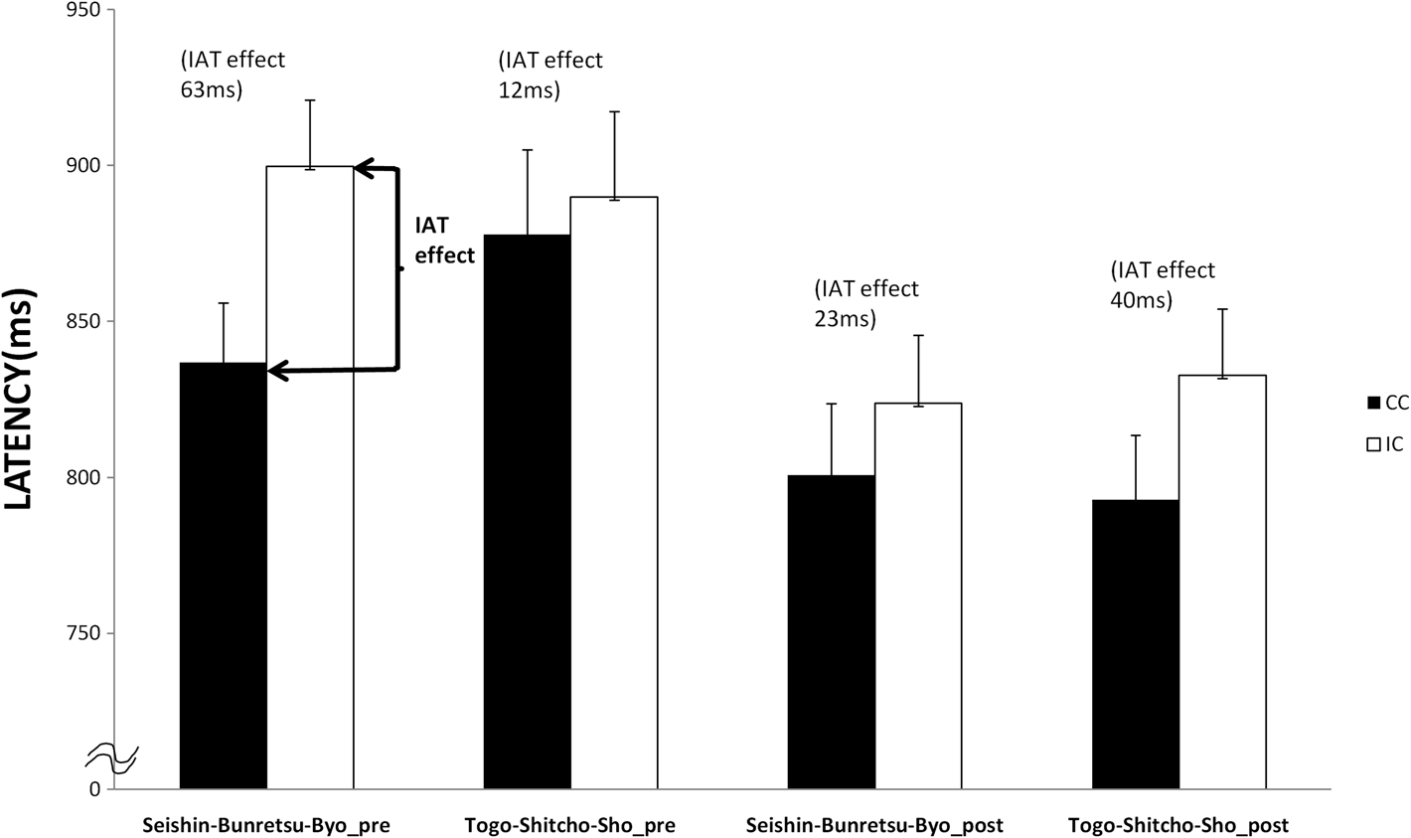
**Before and after clinical training.** Average response latency for CC and IC in the “Seishin-Bunretsu-Byo” version and “Togo-Shitcho-Sho” version of IAT before and after clinical training. ANOVA revealed that response latencies were significantly longer (p<.01) for IC than CC in the old term experiment, not in the new term experiment, before training, and response latencies were significantly longer (p<.05) for IC than CC in the new term experiment, not in the old term experiment, after training.

The average response latencies for CC and IC in the “Seishin-Bunretsu-Byo” and “Togo-Shitcho-Sho” versions of IAT before and after clinical training are shown in Figure 2. Before clinical training for the “Seishin-Bunretsu-Byo” version, the average response latency for CC and IC was 837 ms (SEM=19) and 900 ms (SEM=21), respectively, yielding a 63-ms averaged IAT effect. For the “Togo-Shitcho-Sho” version, average response latency for CC and IC was 878 ms (SEM=27) and 890 ms (SEM=27), respectively, yielding a 12-ms averaged IAT effect.After clinical training for the “Seishin-Bunretsu-Byo” version, average response latency for CC and IC was 801 ms (SEM=22) and 824 ms (SEM=22) respectively, yielding a 23-ms averaged IAT effect. For the “Togo-Shitcho-Sho” version, average response latency for CC and IC was 793 ms (SEM=21) and 833 ms (SEM=21) respectively, yielding a 40-ms averaged IAT effect.3-way ANOVA yielded a significant period main effect, F (1, 50) = 17.2, p<.001, a significant condition main effect, F (1, 50) = 6.9, p<.05, and a significant interaction between term × condition × period, F (1, 50) = 4.52, p<.05. There was no significant term main effect (F (1, 50) = 0.24). There was no significant interaction between term and condition (F (1, 50) = 0.83), between condition and period (F (1, 50) = 0.00), or between period and term (F (1, 50) = 0.15).The significant interaction effect was explored further using a simple main effects analysis, which revealed that response latencies were significantly longer (p<.01) for IC than CC in the old term, not in the new term, before training, and that response latencies were significantly longer (p<.05) for IC than CC in the new term experiment, not in the old term experiment, after training. Other comparisons in the old and new terms did not show any significant differences. There were no other significant main effects or 2- or 3-way interactions. Thus the old term was more congruent with criminal than the new term before clinical training, and the new term, in contrast, was more congruent with criminal than the old term after clinical training.The average total score of Link's devaluation-discrimination scale was 32.41 (S.D. = 4.60) and 32.92 (S.D. = 4.74) before and after, respectively. There were no significant correlations between explicit Link's scale and IAT effect for both the new and old terms before and after clinical training, respectively (r = 0.186, n.s., r = 0.039, n.s., r = 0.182, n.s., r = −0.222, n.s., respectively).

## Discussion

In this study, we assessed the impact of the renaming of the Japanese term for schizophrenia in clinical residents. We also assessed the change in attitudes toward schizophrenia before and after clinical training in psychiatry to elucidate the effect of having contact with schizophrenia patients.The result from before clinical training demonstrated that the old term “Seishin-Bunretsu-Byo” (Mind-Split Disease) was more congruent with criminal than the new term “Togo-Shitcho-Sho” (Integration Disorder), suggesting that the old term was strongly associated with “criminal” vs. “victims”, while the automatic association between the new term and criminal was not strong. This means that our results are consistent with the previous study [22], which indicated that the name change led to reduced stigma, and even for clinical residents, not only for general students.Then, this automatic association between the old term “Seishin-Bunretsu-Byo” and criminal became diminished after clinical psychiatry training, even indicating that the old term had become unfamiliar after clinical training, as the use of the new term “Togo-Shitcho-Sho” in official documents became established in Japan.The most interesting finding from our study, however, was that the new term “Togo-Shitcho-Sho” actually became strongly associated with criminal after clinical psychiatry training. Some previous studies reported the negative and positive attitudes towards schizophrenia held by medical professionals [34,35], but little has been known about how contact with schizophrenia patients affects the formation of negative attitudes towards schizophrenia at implicit and explicit levels. Our result showed that contact with these patients, unexpectedly, changed the attitudes towards schizophrenia of clinical residents in a negative way, indicating that their implicit knowledge structure regarding schizophrenia changed to be strongly associated with criminal, as IAT indirectly measures the combined association strengths of two associative pairs contrasted with the strengths of two other associative pairs. This negative implicit knowledge structure towards schizophrenia does not mean prejudice and stigma. However, as it is detected by implicit measures that predict variation in behavior that is not accounted for by explicit measures such as in conditions where self-presentation concerns are high (i.e., prejudice and stereotyping domains) [41,42], the implicit knowledge structure towards schizophrenia strongly associated with criminal might actually induce discriminative attitudes and behaviors automatically.From an educational standpoint, with the expectation of reducing stigma, certain campaigns have been emphasizing biogenetic explanations of schizophrenia [43]. These kinds of campaigns disseminate the concept that "schizophrenia is an illness like any other" and bring about several effects, such as the causes of mental health problems being attributed to factors outside the self-control of individuals, like biological factors, and people’s attitudes will be less negative and patients and families will experience less blame [44]. On the other hand, it has been argued that biogenetic explanations might cause other complicating issues such as that patients with schizophrenia are viewed as individuals who are unpredictable and dangerous, which then is positively associated with fear [45]. A systematic review of biogenetic causal attributions of mental illness among the general public was conducted by Angermeyer et al. (2011) [14], and it was indicated that increasing public knowledge of the biological and genetic basis was not associated with lesser rejection of people with mental illness, and in fact there seemed to be a danger that biogenetic illness concepts increased rather than decreased public stigma of mental illness. Our findings suggest that a general educational program emphasizing biogenetic explanations of schizophrenia for clinical residents is not sufficient. As pointed out by Corrigan et al. (2004) [46], a popular strategy for combating mental illness may exacerbate other components of stigma, particularly the benevolence and dangerousness stigmas, and they proposed a balanced approach that combats the various myths about mental illness. Based on their findings, more appropriate programs that provide accurate information about violence and schizophrenia, and exposure not only to acute phase patients but also recovered patients who are active in the community would be both beneficial and necessary. Although there has been no established standard educational paradigm for reducing prejudice and stigma, our findings that the implicit knowledge structure regarding schizophrenia was influenced by contact with such patients indicate that appropriate educational programs such as to provide accurate information about violence and schizophrenia for clinical residents would be both beneficial and necessary in the early phase of their clinical training.

The attitudes towards schizophrenia of clinical residents could be influenced not only by contact with schizophrenia patients through their clinical training but also by the attitudes towards schizophrenia of the supervisors of their psychiatry care teams and co-medical staff including psychologists, social workers, and nurses in the psychiatric ward.

We could not find significant differences in Link's scale scores between the new and old terms, nor was there significant correlation between explicit scale and implicit scale for both the new and old terms. Although we do not have precise explanations, self-presentation is considered a cause. IAT is not actually a lie-detector nor does it reveal something that is more true [47], and evidence for the factors moderating the predictive validity of implicit measures is still in its growth phase [41]. IAT is used to evaluate the severity of psychiatric symptoms of various mental illnesses [48-51]. Taken together, IAT appears to be a useful tool for evaluating the attitudes towards mental illness.Our study has some limitations. First, the extent and type of exposure of the clinical residents in this study could not be fully integrated. We could not classify the type of contact they experienced and did not know their previous experience with mental illness, either academically or personally. Also, we assessed only a 1-month clinical training period, not a long-term follow-up, to determine whether changed attitudes towards schizophrenia caused by contact with schizophrenia patients remained thereafter. Thus, the findings may not pertain to other groups. Controlled previous experience and information of the assessment of contact with mental illness would be beneficial for future study. Secondly, mean age of the participants in this study (28.0 years, S.D. = 4.1 years) was older than that of the earlier study (21.5 years, S.D. = 1.4 years) [22]. The possibility of difference in the knowledge level of the old term “Seishin-Bunretsu-Byo” (Mind-Split Disease) cannot be ruled out, since the Japanese term for schizophrenia was changed in 2002. Information concerning prejudice and stigma about mental illness, usually gathered using questionnaires, is subject to response bias due to social desirability [2,23-25], and especially among medical professionals [52]. In order to minimize this bias, we used Link’s scale. However, it remains possible that we cannot directly access explicit attitudes toward schizophrenia using this scale.

Also, we investigated only the association between schizophrenia and criminal using hypertension as control illness by the task of IAT according to Takahashi et al. (2009) [22]. Additional IAT studies on the association between schizophrenia and other stereotypical attributes using different control illnesses are recommended, as also pointed out by Takahashi et al. (2009) [22].

## Conclusions

In this study we found that the renaming of the Japanese term for schizophrenia reduced the negative images of schizophrenia among clinical residents, the same as in non-medical populations, and contact with schizophrenia patients changed the implicit attitudes towards this condition through the clinical training of psychiatry in Japan. Despite some limitations about the extent and type of exposure and accessing explicit attitudes, our results might contribute to an understanding of the formation of negative attitudes toward schizophrenia and could assist in the future development of appropriate clinical training in psychiatry that might reduce prejudice and stigma in respect to schizophrenia. At the same time, the findings merit further investigation of the impact of negative attitudes held by residents on treatment outcomes and rehabilitation of schizophrenia patients.

## Abbreviations

IAT: Implicit Association Test; IC: Incongruent condition; CC: Congruent condition.

## Competing interests

The authors have no competing interests to declare.

## Authors’ contributions

AO, AT, TI and HT designed the study and wrote the protocol. AO, AT, TI and YK managed the literature searches and analyses. AO and TI undertook the statistical analysis, and AO wrote the first draft of the manuscript. All authors read and approved the final manuscript.

## Pre-publication history

The pre-publication history for this paper can be accessed here:

http://www.biomedcentral.com/1471-244X/12/205/prepub
